# White Blood Cell Counts as Risk Markers of Developing Metabolic Syndrome and Its Components in the Predimed Study

**DOI:** 10.1371/journal.pone.0058354

**Published:** 2013-03-19

**Authors:** Nancy Babio, Núria Ibarrola-Jurado, Mònica Bulló, Miguel Ángel Martínez-González, Julia Wärnberg, Itziar Salaverría, Manuel Ortega-Calvo, Ramón Estruch, Lluís Serra-Majem, Maria Isabel Covas, José Vicente Sorli, Jordi Salas-Salvadó, for the PREDIMED Study Investigators

**Affiliations:** 1 Human Nutrition Unit, Sant Joan Hospital, IISPV, Universitat Rovira i Virgili, Reus, Spain; 2 CIBER Fisiopatología de la Obesidad y Nutrición (CIBERobn), Instituto de Salud Carlos III (ISCIII), Reus, Spain; 3 Department of Preventive Medicine and Public Health, Medical School-Clínica, University of Navarra, Pamplona, Spain; 4 Department of Preventive Medicine, University of Málaga, Málaga, Spain; 5 Department of Cardiology, University Hospital Txagorritxu, Vitoria, Spain; Department of Family Medicine, Primary Care Division of Sevilla, Esperanza Macarena Health Center, Sevilla, Spain; 7 Department of Internal Medicine, Hospital Clinic, University of Barcelona, Barcelona, Spain; 8 Department of Clinical Sciences, University of Las Palmas de Gran Canaria, Las Palmas, Spain; 9 Cardiovascular Epidemiology Unit, Municipal Institute for Medical Research (IMIM), Barcelona, Spain; 10 Department of Preventive Medicine and Public Health, School of Medicine, University of Valencia, Valencia, Spain; 11 CIBER Fisiopatología de la Obesidad y Nutricion, Valencia, Spain; University of Las Palmas de Gran Canaria, Spain

## Abstract

**Background:**

The Metabolic Syndrome (MetS) is a cluster of metabolic abnormalities that includes hyperglucemia, hypertension, dyslipidemia and central obesity, conferring an increased risk of cardiovascular disease. The white blood cell (WBC) count has been proposed as a marker for predicting cardiovascular risk. However, few prospective studies have evaluated the relationship between WBC subtypes and risk of MetS.

**Methods:**

Participants were recruited from seven PREDIMED study centers. Both a baseline cross-sectional (n = 4,377) and a prospective assessment (n = 1,637) were performed. Participants with MetS at baseline were excluded from the longitudinal analysis. The median follow-up was 3.9 years. Anthropometric measurements, blood pressure, fasting glucose, lipid profile and WBC counts were assessed at baseline and yearly during the follow-up. Participants were categorized by baseline WBC and its subtype count quartiles. Adjusted logistic regression models were fitted to assess the risk of MetS and its components.

**Results:**

Of the 4,377 participants, 62.6% had MetS at baseline. Compared to the participants in the lowest baseline sex-adjusted quartile of WBC counts, those in the upper quartile showed an increased risk of having MetS (OR, 2.47; 95%CI, 2.03–2.99; P-trend<0.001). This association was also observed for all WBC subtypes, except for basophils. Compared to participants in the lowest quartile, those in the top quartile of leukocyte, neutrophil and lymphocyte count had an increased risk of MetS incidence. Leukocyte and neutrophil count were found to be strongly associated with the MetS components hypertriglyceridemia and low HDL-cholesterol. Likewise, lymphocyte counts were found to be associated with the incidence of the MetS components low HDL-cholesterol and high fasting glucose. An increase in the total WBC during the follow-up was also associated with an increased risk of MetS.

**Conclusions:**

Total WBC counts, and some subtypes, were positively associated with MetS as well as hypertriglyceridemia, low HDL-cholesterol and high fasting glucose, all components of MetS.

**Trial registration:**

Controlled-Trials.comISRCTN35739639.

## Introduction

In recent years, the prevalence of metabolic syndrome (MetS) has increased dramatically in both developed and developing countries. MetS is a constellation of cardiovascular risk factors (impaired glucose tolerance, hypertension, dyslipidemia and central obesity) strongly associated with type 2 diabetes, cardiovascular diseases, and other chronic conditions, all of which increase the risk of mortality [1], [2]. Thus, early identification of the subjects who are at high risk of developing MetS may help prevent associated cardiovascular events.

The peripheral circulating white blood cell (WBC) count is an objective marker of acute infection, tissue damage, and other inflammatory conditions. Moderately high levels of leukocyte counts have been positively associated with type 2 diabetes [3], [4] and the progression or severity of atherosclerosis [5], [6]. Thus, the leukocyte count has been proposed as an emerging biomarker for predicting future cardiovascular events [7], [8] and mortality [9]. The assessment of the association between the WBC count and the development of the MetS may help to a better understanding of the pathophysiology of the MetS because chronic subclinical inflammation has been implicated in the genesis of MetS, [10], [11] and the leukocyte count may be seen as an inflammation marker that is related to this condition. In fact, for over a decade cross-sectional studies have been showing that there is a close relationship between WBC and MetS prevalence, mainly in Asian populations [4], [12]–[20]. Considerably less research, however, has explored the associations between MetS or its features and different subtypes of leukocyte [14]–[19], [21].

The prospective studies that have examined the relationship between WBC count and the risk of MetS are limited and controversial. To the best of our knowledge, only two studies have explored these associations. One of them was a cohort of preadolescents, adolescents and young adults that longitudinally analyzed the association between WBC count and MetS incidence in white individuals [22]. Recently, an increase was also observed in the MetS incidence across WBC count quartiles in a cohort of workers followed for 7 years, [23] but adiposity indexes were not taken into account when this association was explored. Beyond these two studies, the associations between leukocyte subtype count and MetS (or its components) have never been explored prospectively.

Therefore, the aim of the present study was to evaluate total leukocyte count and subtype count as possible predictors of the development of MetS and/or its features in a population at high risk of cardiovascular disease.

## Methods

### Design and population

The PREDIMED (Prevención con DIeta MEDiterránea) Study is a large, parallel-group, multicenter, randomized, controlled clinical trial that aims to assess the effects of the Mediterranean Diet (MedDiet) on the primary prevention of cardiovascular disease (http://www.predimed.es; http://www.predimed.org). The design, methodology and eligibility criteria for the PREDIMED study have been previously described [24], [25]. Briefly, 7,447 community-dwelling men (aged 55–80 years) and women (aged 60–80 years) with no previously documented cardiovascular disease were recruited. They were eligible if they had type 2 diabetes, or three or more of the following cardiovascular risk factors: hypertension, high plasma LDL-cholesterol, low plasma HDL-cholesterol, overweight or obesity, current smoking, or a family history of premature coronary heart disease. Participants were randomized to three intervention groups (two of which were advised to follow a MedDiet (MedDiet) supplemented with either virgin olive oil or nuts, and one of which was advised to follow a low-fat diet).

In the present study, we cross-sectionally and longitudinally analyzed the associations between WBC counts and the presence and development of MetS and its components in a subset of 4,377 participants recruited in seven PREDIMED centers where information about WBC count was collected (Navarra, Málaga, Barcelona-North, Vitoria, Sevilla, Gran Canaria, and Reus). No significant differences were found for the main baseline variables (age, sex and BMI) between PREDIMED participants included or not included in the present analysis. Only those participants free of MetS at baseline were included in the longitudinal analysis. In the longitudinal analysis, we used as exposure the baseline leukocyte count (or the leukocyte subtypes). The main outcome was the incidence of the MetS during any assessment along all the follow-up period, expanding up to 7 years (median follow-up: 3.9 years). Therefore any new case of MetS occurring in any time point during the follow-up period among subjects initially free of the MetS was considered as an incident event.

### Ethics statement

All participants provided informed consent. The Institutional Review Board (IRB) of the Hospital Clinic (Barcelona, Spain) accredited by the US Department of Health and Human Services (DHHS) update for Federal wide Assurance for the Protection of Human Subjects for International (Non-US) Institutions # 00000738 approved the study protocol on July, 16, 2002. The protocol was also approved by the respective institutional ethical review boards.

### Outcomes

MetS was defined in accordance with the updated harmonized criteria of the International Diabetes Federation and the American Heart Association/National Heart, Lung, and Blood Institute [26]. That is to say subjects were regarded as having MetS when they had three or more of the following components:

- Elevated waist circumference for European individuals (≥88 cm and ≥102 cm in women and men, respectively).
- Elevated triglycerides [150 mg/dL (1.7 mmol/L)] or drug treatment for elevated triglycerides.
- Low concentrations of high density lipoprotein cholesterol (HDL-c) [40 mg/dL (1.0 mmol/L) in men; 50 mg/dL (1.3 mmol/L) in women] or drug treatment for low HDL-C.
- Elevated blood pressure (systolic ≥130 and/or diastolic ≥85 mm Hg) or antihypertensive drug treatment.
- Elevated fasting glucose [≥100 mg/dL (5.5 mmol/L)] or drug treatment for elevated glucose.

### Measurements

In a personal interview, participants completed: a) a questionnaire about lifestyle variables, medical history and medication use; b) a 14-item validated questionnaire [27] designed to assess the degree of adherence to the MedDiet; and, c) the validated Spanish version [28] of the Minnesota Leisure-Time Physical Activity Questionnaire. Trained personnel took anthropometric measurements (weight, height and waist circumference) and blood pressure was measured using a validated oscillometer [Omron HEM705CP, Hoofddorp, Netherlands] in triplicate. These questionnaires were administered and measurements were recorded at baseline and yearly during follow-up.

### Biochemical determinations

Fasting plasma glucose, serum total cholesterol, HDL-cholesterol and triglyceride concentrations were measured at baseline and yearly, using standard enzymatic automated methods. LDL-cholesterol was estimated by the Friedewald equation when the direct parameter was not available. WBC and different leukocyte counts (neutrophil, lymphocyte, monocyte, basophils, and eosinophil) were measured using an automated analyzer.

### Statistical analyses

Continuous variables are presented as median and interquartile range [IR] for non-normally distributed data. Data with skewed distributions determined with the Kolmogorov-Smirnov test were log-transformed before analysis.

Partial correlation coefficients adjusted for sex were used to analyze the relationship between the total leukocyte count (or subtypes) and each of the specific criteria for the MetS (abdominal obesity, hypertriglyceridemia, low HDL-cholesterol levels, hypertension, and high fasting plasma glucose concentrations).

To assess the risk of MetS for each category of WBC count in cross-sectional analyses, participants were categorized according to their baseline sex-adjusted WBC count quartiles. To prospectively assess the risk of MetS or its components, new WBC count quartiles were constructed for participants free of MetS at baseline. Unadjusted and adjusted logistic regression models were fitted to assess the associations between baseline quartiles of total WBC count (or each leukocyte subtype) and the prevalence of MetS. Logistic regression models were adjusted for the intervention group (two dummy variables), baseline age (years), smoking (three categories), leisure-time physical activity expressed in metabolic equivalents (METs-min/d), MedDiet score (0–14 points), alcohol consumption (g/day), BMI (kg/m^2^), and recruitment center.

Unadjusted and adjusted logistic regression models were also fitted to longitudinally assess the above associations. In the longitudinal analysis we also used as exposure the quartiles of baseline WBC (or each leukocyte subtype count). The outcome was the incidence of MetS (or each of the specific defining criteria). In the adjusted models, the following potential confounders were included as covariates: intervention group, baseline age, tobacco use, physical activity, MedDiet score and alcohol consumption (Model 1); the same confounders used in model 1 plus baseline BMI and recruitment centre (Model 2), and the same confounders used in previous models plus baseline features of the MetS (dichotomous variables) [the fully-adjusted model]. To assess linear trends in all logistic regression models we introduced the median value of each quartile of the WBC count as a continuous variable in the different models.

To estimate the change in the probability of the MetS being diagnosed given that two years ago the leukocyte count differed by a certain amount, we fitted generalized estimating equations with robust standard errors, to estimate the odds ratio of being classified as having the MetS given that two years ago the leukocyte count differed by 2*10^9^ cells/L (an amount close to the standard deviation). We adjusted these estimates for the same confounders as the main logistic regression model and thus obtained an outer expectation for all the available time points, together with an inner expectation for all possible values of the confounders.

We analyzed the area under the ROC curve to predict the prevalence or the incidence of MetS with and without the variable leukocyte count (or leukocyte subtypes). We compared ROC curves using Stata 12.0’s roccomp command, which uses the method suggested by DeLong, DeLong, and Clarke-Pearson [29].

Cox regression models were fitted to assess the relative risk of MetS across total leukocyte, neutrophil and lymphocyte quartiles, estimated with hazard ratios (HR) and 95% confidence intervals (CI). The time variable used was the interval between the baseline measurement date and the date of the last follow-up, death or outcome diagnosis, whichever occurred first. Models were adjusted for the same variables described for model 2.

A second longitudinal analysis was conducted to assess the associations between changes in WBC count during follow-up and the incidence of MetS. The participants were dichotomized in two categories: a) those who remained in the same WBC count (leukocyte, neutrophil and lymphocyte) quartiles (2^nd^, 3^rd^, or 4^th^) or who switched to a higher quartile during the follow-up; and b) those who remained in the 1^st^ quartile or switched to a lower quartile during the follow-up.

Two-tailed P-values <0.05 were considered significant. Statistical analyses were done with the SPSS (version 19.0, SPSSInc, Chicago, IL).

## Results

Table 1 classifies the participants according to their baseline WBC count (quartiles). Of the 4,377 participants, 62.6% (n = 2,740) met the criteria for MetS at baseline. For waist circumference the criteria for MetS were met in 72%, for hypertriglyceridemia in 33%, for low HDL-c in 27%, for elevated blood pressure in 94.4%, and for elevated fasting glucose components of the MetS in 64.6%.

**Table 1:** Baseline characteristics of participants across sex-adjusted leukocyte count quartiles (first to fourth: Q1 to Q4).

	*Q1*	*Q2*	*Q3*	*Q4*	*P-value* a
Participants, n	1085	1113	1069	1110	
Leukocyte counts, cells x 10^9^/L - median [percentile 25^th^–75^th^]	4.74 [4.30–5.06]	5.84 [5.60–6.10]	6.90 [6.60–7.15]	8.50 [7.90–9.40]	NA
Age, years - mean ± SD	67.2 ± 6.1	67.1 ± 6.1	67.1 ± 6.2	66.9 ± 6.3	0.711
Sex,					
Women, % (n)	57.1 (619)	55.7 (620)	57.7 (617)	56.3 (625)	NA
Body Mass Index, kg/m^2^ - mean ± SD	29.5 ± 3.7	29.9 ± 3.5	30.1 ± 3.7	30.3 ± 3.9	<0.001
Waist circumference, cm - mean ± SD	98.6 (10.1)	99.5 (10.4)	100.4 (10.0)	101.1 (10.2)	<0.001
Current smoker, % (n)	7.8 (85)	11.1 (124)	15.2 (163)	24.1 (268)	<0.001
Former smoker, % (n)	24.1 (262)	27.9 (310)	25.4 (271)	23.2 (257)	0.064
Physical activity, METs - mean ± SD	263 ± 267	246 ± 242	247 ± 267	218 ± 225	< 0.001
Mediterranean diet score, 0 to 14 points - mean ± SD	8.8 ± 1.9	8.7 ± 2.0	8.7 ± 1.9	8.6 ± 1.9	0.052
Systolic blood pressure, mmHg - mean ± SD	147.6 ± 19.3	148.9 ± 20.2	150.0 ± 20.5	151.4 ± 20.9	<0.001
Diastolic blood pressure, mmHg - mean ± SD	82.4 ± 10.9	83.2 ±10.5	83.1 ± 10.4	83.8 ± 11.1	0.026
Fasting plasma glucose, mg/dL - mean ± SD	114.0 ± 35.7	120.0 ± 40.7	124.0 ± 42.0	127.1 ± 45.8	<0.001
Triglycerides, mg/dL [percentile 25^th^-75^th^]	104.0 [77.0–142.0]	120.0 [88.0–163.0]	124.5 [94.0–168.0]	132.0 [100.0–178.7]	<0.001
HDL-cholesterol, mg/dL [percentile 25^th^–75^th^]	55.0 [47.0–67.0]	53.0 [45.0–62.9]	52.0 [44.0–62.0]	59.0 [43.0–59.0]	<0.001
Prevalence of type 2 diabetes mellitus, % (n)	40.1 (435)	44.0 (490)	51.2 (547)	52.0 (577)	<0.001
Metabolic syndrome and prevalence of its components					
Metabolic syndrome, % (n)	51.7 (561)	60.1 (669)	65.7 (702)	71.8 (808)	<0.001
Abdominal obesity, % (n)	68.6 (737)	71.7 (791)	74.9 (796)	74.9 (828)	0.002
Hypertriglyceridemia, % (n)	24.1 (261)	32.9 (365)	34.6 (369)	40.5 (448)	<0.001
Low HDL-cholesterol level, % (n)	21.2 (229)	25.8 (286)	28.6 (305)	32.9 (362)	<0.001
Hypertension, % (n)	93.5 (1015)	94.0 (1045)	94.0 (1005)	96.3 (1067)	0.020
High fasting plasma glucose concentrations, % (n)	56.8 (616)	63.0 (701)	67.4 (720)	71.2 (790)	<0.001

a ANOVA for continuous variables and χ^2^ for categorical variables test were used for comparisons across the total leucocyte count quartiles.

Participants with MetS had higher peripheral leukocyte counts (median 6.5, IR [5.5–7.7] cells x10^9^/L) than those without MetS (6.0 [5.1–7.0]; P <0.001). Mean body-mass-index, waist circumference, blood pressure, and fasting glucose and triglyceride concentrations were significantly higher in the upper quartiles than in the lowest (reference). The percentage of participants with MetS or each of the defining criteria for the MetS was significantly higher across successively increasing WBC count quartiles.

After adjusting for sex, low but significant direct correlations were found between log-transformed leukocyte, neutrophil or lymphocyte counts and plasma glucose (0.116, 0.117 and 0.042, respectively), triglycerides (0.178, 0.131 and 0.177, respectively), and waist circumference (0.094, 0.93 and 0.034, respectively). Conversely, significant inverse correlations were observed between the cell counts and log-transformed HDL-c concentrations (–0.144, –0.135 and –0.084, respectively).

In the cross-sectional baseline analysis, participants in the upper quartile of total leukocyte count had a greater prevalence of MetS than participants in the lowest quartile (OR, 2.47; 95% CI, 2.03–2.99; P for trend <0.001). This significant association was also observed for the neutrophil count (OR, 2.09; 95% CI, 1.73–2.54; P for trend <0.001), lymphocytes (OR, 1.56; 95% CI, 1.29–1.88; P for trend <0.001), monocytes (OR, 1.41; 95% CI, 1.16–1.72; P for trend <0.001), and eosinophils (OR, 1.43; 95% CI, 1.18–1.72; P for trend <0.001) (Table 2). These associations remained significant even after participants with WBC count ≥11.0 cells x 10^9^/L (n = 83, 1.9% of the total sample) were excluded.

**Table 2:** Prevalence of metabolic syndrome for sex-adjusted quartiles of total white blood cell count and leukocyte subtypes.

	*Q1* *n = 1085*	*Q2* *n = 1113*	*Q3* *n = 1069*	*Q4* *n = 1110*	*P for trend*
***Total leukocytes***					
Cells x 10^9^/L- median [percentile 25^th^–75^th^]	4.74 [4.30–5.06]	5.84 [5.60–6.10]	6.90 [6.60–7.15]	8.50 [7.90–9.40]	NA
Unadjusted	1	1.41 (1.19–1.67)	1.79 (1.50–2.13)	2.29 (1.91–2.73)	< 0.001
Adjusted^a^	1	1.35 (1.12–1.61)	1.70 (1.42–2.05)	2.47 (2.03–2.99)	< 0.001
***Neutrophils***					
Cells x 10^9^/L- median [percentile 25^th^–75^th^]	2.53 [2.20–2.89]	3.22 [2.86–3.60]	3.90 [3.52–4.34]	5.12 [4.43–5.90]	NA
Unadjusted	1	1.23 (1.03–1.45)	1.65 (1.39–1.96)	2.21 (1.85–2.64)	< 0.001
Adjusted^a^	1	1.22 (1.02–1.47)	1.66 (1.38–2.00)	2.09 (1.73–2.54)	< 0.001
***Lymphocytes***					
Cells x 10^9^/L- median [percentile 25^th^–75^th^]	1.54 [1.32–1.81]	1.95 [1.66–2.27]	2.19 [1.82–2.53]	2.55 [2.09–3.07]	NA
Unadjusted	1	1.20 (1.01–1.43)	1.44 (1.21–1.71)	1.59 (1.34–1.89)	< 0.001
Adjusted^a^	1	1.10 (0.92–1.33)	1.41 (1.17–1.70)	1.56 (1.29–1.88)	< 0.001
***Monocytes***					
Cells x 10^9^/L- median [percentile 25^th^–75^th^]	0.34 [0.27–0.42]	0.42 [0.34–0.50]	0.48 [0.38–0.58]	0.59 [0.46–0.71]	NA
Unadjusted	1	1.17 (0.98–1.39)	1.16 (0.97–1.37)	1.37 (1.15–1.63)	0.001
Adjusted^a^	1	1.22 (1.01–1.47)	1.20 (0.99–1.46)	1.41 (1.16–1.72)	0.002
***Eosinophils***					
Cells x 10^9^/L- median [percentile 25^th^–75^th^]	0.12 [0.08–0.17]	0.15 [0.10–0.21]	0.18 [0.12–0.25]	0.21 [0.14–0.30]	NA
Unadjusted	1	1.31 (1.10–1.56)	1.46 (1.23–1.74)	1.46 (1.23–1.74)	< 0.001
Adjusted^a^	1	1.33 (1.11–1.60)	1.43 (1.19–1.72)	1.43 (1.18–1.72)	< 0.001
***Basophils***					
Cells x 10^9^/L- median [percentile 25^th^–75^th^]	0.03 [0.02–0.04]	0.03 [0.02–0.04]	0.04 [0.02–0.05]	0.04 [0.03–0.06]	NA
Unadjusted	1	1.02 (0.86–1.22)	0.93 (0.78–1.10)	1.11 (0.93–1.32)	0.470
Adjusted^a^	1	1.06 (0.88–1.27)	0.95 (0.79–1.14)	1.10 (0.91–1.32)	0.571

Odds ratios (95% confidence intervals) with the lowest quartile as the reference category.

a Logistic regression models adjusted for intervention group, and baseline age, BMI, tobacco use, physical activity, Mediterranean diet score, alcohol consumption and study centre; NA: Not applicable.

**Figure 1:**
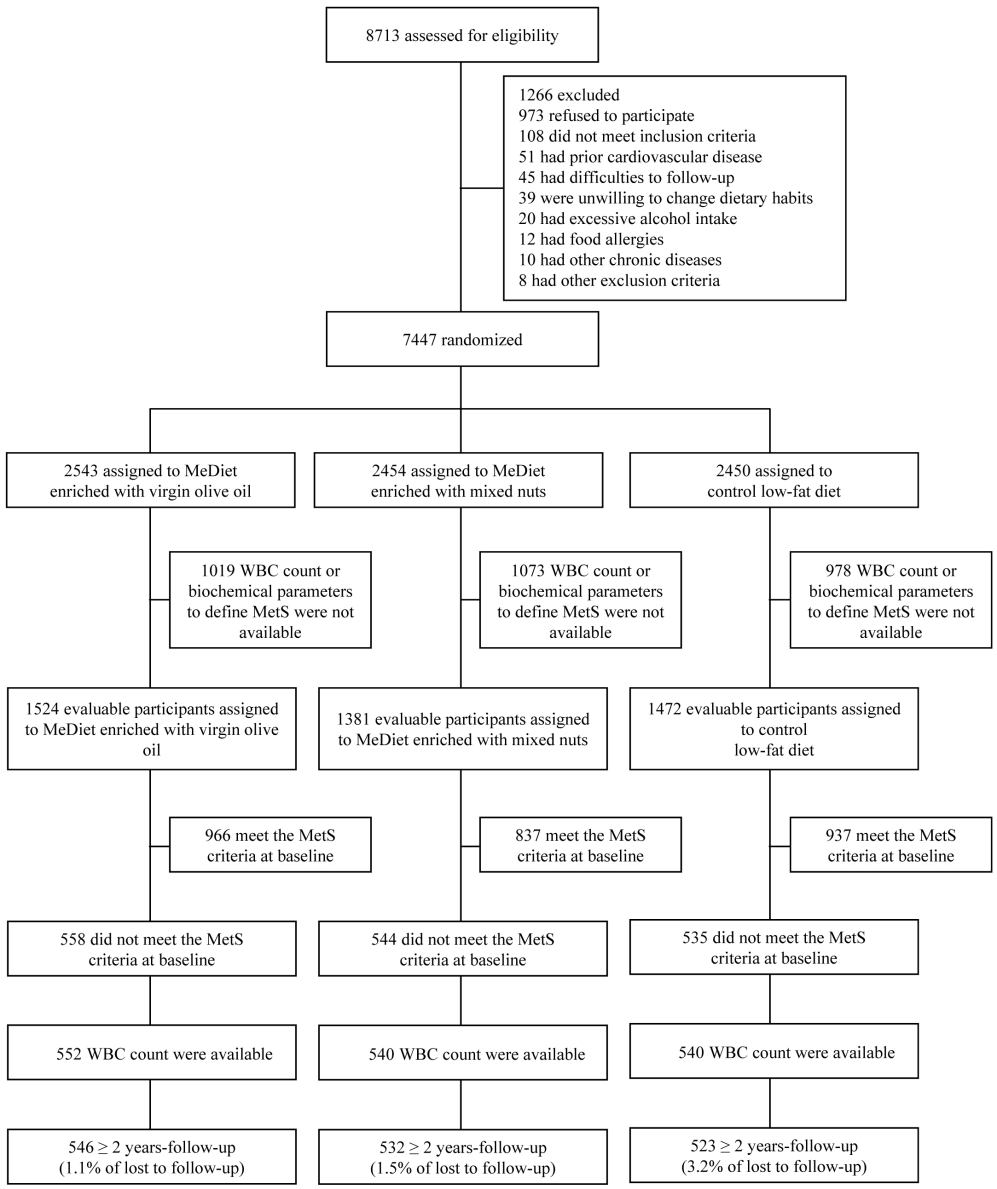
Flowchart of study participants. Abbreviations: MedDiet = Mediterranean diet; MetS =  Metabolic syndrome; WBC =  white blood cell.

Of the 4,377 participants, 1,637 did not meet the MetS criteria at baseline. For 16 of them, enough data were available to diagnose metS at baseline. Thus, longitudinal analyses were carried out in a sample of 1,621 individuals (546, 532 and 523 participants randomized to a MedDiet supplemented with virgin olive oil, a MedDiet supplemented with nuts, and a low-fat control diet, respectively). The retention rates for ≥ 2 years follow-up were 98.9%, 98.5% and 96.8%, respectively) (see figure 1.)

Over the 7-year study period, with a median follow-up of 3.9 [2.0–5.8] years, 726 (350 men and 376 women) of the 1,621 participants free of MetS at baseline developed new-onset MetS (cumulative incidence: 44.8%, 95% CI 42.4–47.2). Participants who developed MetS had significantly higher WBC counts at baseline than those who did not (6.1 [5.1–7.3] cells x 10^9^/L *vs*. 5.9 [5.0–7.0] cells x 10^9^/L, P  = 0.003). The incidence of MetS was significantly higher across leukocyte and neutrophil quartiles, and of borderline statistical significance across lymphocyte quartiles (P  = 0.072).

Table 3 shows the logistic regression analyses that explore the associations between total leukocytes or other subtypes of WBC count and incidence of MetS. Compared to subjects in the lowest quartile, subjects in the highest quartile of leukocyte, neutrophil and lymphocyte counts had an increased risk of MetS incidence. In the fully-adjusted model these associations remained significant for neutrophil counts, and were of borderline significance for total leukocytes (P  = 0.065) and lymphocytes (P  = 0.090). Considering that there was a difference in the leukocyte count of 2*10^9^ cells/L two years before the MetS was assessed, the estimates for the Odds Ratio (OR) obtained using generalized estimating equations with robust standard errors (Appendix S1) were 1.20 (95%CI, 1.08–1.34) in the multivariable adjusted model (P = 0.001), and 1.17 (95%CI, 1.05–1.31) in the additionally adjusted model for baseline criteria for MetS. The Hosmer-Lemeshow test of the multivariable adjusted logistic regression model using leukocyte quartiles as predictors of the incidence of MetS during follow-up showed a reasonable goodness of fit (P  =  0.81). The results were very similar when individuals (n = 24) with increased leukocyte counts (>11*10^9^ cells/L) were excluded from the analysis (Appendix S1). The area under the ROC curve was only slightly higher (AUC = 0.609) when the total leukocyte count was used than with the basic model (AUC = 0.603). There was no significant difference between them (P =  0.29).

**Table 3:** Incidence of new cases of metabolic syndrome during the follow-up among subjects initially free of metabolic syndrome according to baseline sex-adjusted leukocyte counts.

	*Q1*n = 399	*Q2*n = 409	*Q3*n = 410	*Q4*n = 399	*P-value*
***Total leukocytes***					
Metabolic syndrome incidence, %	39.0	45.1	45.0	50.0	0.020^a^
Unadjusted	1	1.29 (0.97–1.70)	1.28 (0.97–1.69)	1.56 (1.18–2.07)	0.003^b^
Adjusted model 1^c^	1	1.28 (0.97–1.69)	1.26 (0.95–1.67)	1.54 (1.15–2.05)	0.006^b^
Adjusted model 2^d^	1	1.22 (0.92–1.63)	1.22 (0.92–1.62)	1.53 (1.12–2.02)	0.009^b^
Fully-adjusted model^e^	1	1.19 (0.88–1.59)	1.14 (0.85–1.53)	1.37 (1.01–1.86)	0.062^b^
***Neutrophils***					
Metabolic syndrome incidence, %	41.4	40.5	44.4	57.7	0.005^a^
Unadjusted	1	0.97 (0.73–1.28)	1.13 (0.86–1.50)	1.52 (1.15–2.01)	<0.001^b^
Adjusted model1^c^	1	0.95 (0.85–1.48)	1.12 (0.85–1.48)	1.49 (1.12–2.00)	0.001^b^
Adjusted model 2^d^	1	0.97 (0.73–1.29)	1.12 (0.84–1.49)	1.47 (1.09–1.96)	0.002^b^
Fully-adjusted model^e^	1	0.89 (0.66–1.20)	1.04 (0.77–1.39)	1.32 (0.98–1.80)	0.004^b^
***Lymphocytes***					
Metabolic syndrome incidence, %	40.6	41.9	48.3	47.1	0.072^a^
Unadjusted	1	1.06 (0.80–1.40)	1.37 (1.03–1.81)	1.30 (0.99–1.73)	0.025^b^
Adjusted model 1^c^	1	1.05 (0.79–1.43)	1.36 (1.03–1.81)	1.27 (0.96–1.69)	0.043^b^
Adjusted model 2^d^	1	1.02 (0.77–1.36)	1.31 (0.99–1.75)	1.29 (0.96–1.72)	0.039^b^
Fully-adjusted model^e^	1	1.05 (0.78–1.42)	1.28 (0.95––1.72)	1.24 (0.91–1.67)	0.106^b^

Odds ratios (95% confidence intervals).

a P-value by χ^2^ test.

b P for trend.

c Logistic regression model adjusted for intervention group, and baseline age, tobacco use, physical activity, Mediterranean diet score and alcohol consumption.

d Logistic regression models adjusted for variables used in model 1 plus baseline BMI and recruitment centre.

e Logistic regression models adjusted for variables used in model 2 plus baseline features of the metabolic syndrome.

Cox regression showed that participants in the highest quartile of leukocyte and neutrophil counts showed a significantly high risk of MetS than those in the lowest quartile (Hazard ratio [HR] for total leukocyte count 1.34 [95% CI, 1.08–1.66], P_Q4 *vs* Q1_  = 0.009; HR for neutrophil count 1.39 [95% CI, 1.13–1.72], P_Q4 *vs* Q1_  = 0.002). No significant association was observed between the lymphocyte count and the risk of MetS (data not shown).

Table 4 shows the incidence of each of the specific MetS defining criteria in subjects initially free of the respective component for each of the total leukocyte, neutrophil and lymphocyte count quartiles. The incidence of hypertriglyceridemia and low HDL-cholesterol increased significantly across leukocyte count quartiles.

**Table 4:** Incidence of each metabolic syndrome component among subjects initially free of its respective component according to baseline sex–adjusted leukocyte counts. Odds ratios (95% confidence intervals).

	*Q1*	*Q2*	*Q3*	*Q4*	*P-value*
**High fasting plasma glucose**					
***Leukocyte***					
Participants, n	**371**	**383**	**365**	**368**	
High fasting plasma glucose incidence, %	37.3	38.3	36.5	36.8	0.066^a^
Unadjusted	1	0.96 (0.71–1.30)	1.28 (0.95–1.71)	1.33 (0.99–1.71)	0.017^b^
Adjusted model 1^c^	1	0.95 (0.70–1.28)	1.24 (0.92–1.68)	1.30 (0.96–1.77)	0.033^b^
Adjusted model 2^d^	1	0.91 (0.67–1.24)	1.20 (0.88–1.02)	1.25 (0.92–1.71)	0.058^b^
***Neutrophils***					
Unadjusted	1	0.94 (0.70–1.27)	1.32 (0.98–1.77)	1.18 (0.88–1.59)	0.058^b^
Adjusted model 1^c^	1	0.93 (0.69–1.26)	1.29 (0.96–1.74)	1.14 (0.84–1.55)	0.085^b^
Adjusted model 2^d^	1	0.95 (0.70–1.29)	1.33 (0.98–1.80)	1.16 (0.85–1.58)	0.121^b^
***Lymphocytes***					
Unadjusted	1	0.93 (0.69–1.26)	1.47 (1.09–1.98)	1.45(1.07–1.95)	0.001^b^
Adjusted model 1^c^	1	0.92 (0.68–1.25)	1.43 (1.06–1.94)	1.40 (1.03–2.90)	0.003^b^
Adjusted model 2^d^	1	0.90 (0.66–1.23)	1.38 (1.02–1.87)	1.34 (0.99–1.82)	0.009^b^
**Hypertriglyceridemia**					
***Leukocyte***					
Participants, n	**742**	**734**	**745**	**730**	
Hypertriglyceridemia incidence, %	25.9	28.7	31.0	35.1	0.001^a^
Unadjusted	1	1.15 (0.92–1.45)	1.29 (1.03–1.62)	1.55 (1.24–1.94)	<0.001^b^
Adjusted model 1^c^	1	1.23 (0.92–1.46)	1.28 (1.02–1.61)	1.52 (1.21–1.91)	<0.001^b^
Adjusted model 2^d^	1	1.21 (0.90–1.43)	1.25 (1.00–1.58)	1.48 (1.17–1.86)	0.001^b^
***Neutrophils***					
Unadjusted	1	1.18 (0.93–1.48)	1.28 (1.02–1.61)	1.45 (1.16–1.82)	0.001^b^
Adjusted model 1^c^	1	1.17 (0.93–1.48)	1.29 (1.02–1.62)	1.43 (1.14–1.81)	0.002^b^
Adjusted model 2^d^	1	1.19 (0.95–1.50)	1.46 (1.16–1.84)	1.48 (1.17–1.88)	0.002^b^
***Lymphocytes***					
Unadjusted	1	1.07 (0.85–1.36)	1.33 (1.06–1.67)	1.46 (1.16–1.84)	<0.001^b^
Adjusted model 1^c^	1	1.06 (0.84–1.34)	1.29 (1.02–1.62)	1.39 (1.12–1.74)	0.001^b^
Adjusted model 2^d^	1	1.02 (0.80–1.29)	1.21 (0.96–1.53)	1.33 (1.05–1.67)	0.006^b^
**Low HDL-cholesterol**					
***Leukocyte***					
Participants, n	**825**	**756**	**820**	**795**	
Low-HDL cholesterol incidence, %	21.8	27.3	27.5	32.3	<0.001^a^
Unadjusted	1	1.35 (1.07–1.70)	1.36 (1.08–1.71)	1.71 (1.37–2.14)	< 0.001^b^
Adjusted model 1^c^	1	1.36 (1.08–1.72)	1.38 (1.10–1.73)	1.79 (1.42–2.25)	< 0.001^b^
Adjusted model 2^d^	1	1.35 (1.08–1.71)	1.36 (1.08–1.71)	1.74 (1.39–2.19)	< 0.001^b^
***Neutrophils***					
Unadjusted	1	1.09 (0.86–1.38)	1.57 (1.25–1.97)	1.72 (1.37–2.16)	<0.001^b^
Adjusted model 1^c^	1	1.10 (0.87–1.40)	1.60 (1.27–2.01)	1.79 (1.42–2.25)	<0.001^b^
Adjusted model 2^d^	1	1.10 (0.87–1.40)	1.57 (1.25–1.98)	1.74 (1.39–2.19)	<0.001^b^
***Lymphocytes***					
Unadjusted	1	1.09 (0.82–1.29)	1.06 (0.85–1.33)	1.06 (0.85–1.33)	0.559^b^
Adjusted model 1^c^	1	1.03 (0.85–1.34)	1.07 (0.84–1.33)	1.06 (0.84–1.33)	0.581^b^
Adjusted model 2^d^	1	1.03 (0.82–1.29)	1.07 (0.85–1.34)	1.06 (0.84–1.30)	0.583^b^
**High blood pressure**					
***Leukocyte***					
Participants, n	**58**	**61**	**63**	**61**	
High blood pressure incidence, %	78.9	78.3	79.4	71.7	0.718^a^
Unadjusted	1	0.96 (0.40–2.33)	1.03 (0.43–2.48)	0.89 (0.33–2.39)	0.415^b^
Adjusted model 1^c^	1	1.16 (0.46–2.93)	1.13 (0.45–2.84)	0.80 (0.32–1.96)	0.621^b^
Adjusted model 2^d^	1	0.83 (0.47–3.04)	1.09 (0.43–2.76)	0.81 (0.33–2.02)	0.625^b^
***Neutrophils***					
Unadjusted	1	0.42 (0.17–1.03)	0.47 (0.18–1.19)	0.79 (0.30–2.06)	0.696^b^
Adjusted model 1^c^	1	0.40 (0.16–1.05)	0.48 (0.19–1.22)	0.83 (0.30–2.28)	0.789^b^
Adjusted model 2^d^	1	0.39 (0.15–1.02)	0.90 (0.37–3.19)	0.87 (0.30–2.45)	0.900^b^
***Lymphocytes***					
Unadjusted	1	0.94 (0.39–2.28)	0.90 (0.37–3.19)	0.66 (0.28–1.54)	0.347^b^
Adjusted model 1^c^	1	1.00 (0.40–2.49)	1.15 (0.45–2.90)	0.76 (0.32–1.85)	0.642^b^
Adjusted model 2^d^	1	0.93 (0.37–2.36)	1.08 (0.42–2.79)	0.67 (0.27–1.68)	0.487^b^
**Abdominal obesity**					
***Leukocyte***					
Participants, n	**302**	**294**	**303**	**300**	
Abdominal obesity incidence, %	37.3	40.7	36.6	39.5	0.716^a^
Unadjusted	1	1.15 (0.82–1.60)	0.97 (0.69–1.35)	1.10 (0.79–1.53)	0.844^b^
Adjusted model 1^c^	1	1.15 (0.83–1.62)	0.99 (0.66–1.39)	1.18 (0.84–1.66)	0.782^b^
Adjusted model 2^d^	1	1.08 (0.76–1.54)	1.02 (0.67–1.46)	1.20 (0.84–1.73)	0.398^b^
***Neutrophils***					
Unadjusted	1	0.89 (0.64–1.24)	0.82 (0.58–1.14)	1.01 (0.73–1.41)	0.932^b^
Adjusted model 1^c^	1	0.89 (0.64–1.26)	0.91(0.57– 1.14)	1.02 (0.72–1.45)	0.956^b^
Adjusted model 2^d^	1	1.00 (0.66–1.35)	0.90 (0.63–1.29)	1.13 (0.79–1.62)	0.594^b^
***Lymphocytes***					
Unadjusted	1	1.21 (0.87–1.69)	1.19 (0.85–1.66)	0.98 (0.70–1.37)	0.855^b^
Adjusted model 1^c^	1	1.26 (0.87–1.77)	1.25 (0.89–1.76)	1.03 (0.72–1.35)	0.882^b^
Adjusted model 2^d^	1	1.22 (0.81–1.74)	1.17 (0.82–1.67)	0.93 (0.65–1.35)	0.669^b^

a P-value =  χ^2^ test; ^b^ P for trend; ^c^ Logistic regression models adjusted for intervention group, and baseline variables: age, current and former smoker, physical activity, Mediterranean diet score and alcohol; ^d^ Logistic regression model adjusted for model 1 plus baseline BMI and recruitment node.

Participants initially free of hypertriglyceridemia at baseline and who were in the upper quartile of leukocyte, neutrophil and lymphocyte counts had a significantly higher risk of developing the hypertriglyceridemia criteria of the MetS during the follow-up even after adjusting for all potential confounders.

Participants free of the MetS low-HDL cholesterol criteria at baseline and who were in the upper quartile of the total leukocyte and neutrophil counts also had a significantly higher risk of developing this MetS component during the follow-up even after adjusting for all confounders.

For high fasting plasma glucose the same positive association was shown across leukocyte and lymphocyte quartiles, but the association across neutrophil quartiles was not statistically significant (P  = 0.121).

Finally, those participants who remained in the same WBC count quartiles (2^nd^, 3^rd^, or 4^th^) or switched to a higher quartile during the follow-up had a 32% higher risk of developing MetS than those participants who remained in the 1^st^ quartile or switched to a lower quartile during the follow-up (Table 5). This association remained significant even after additionally adjusting for baseline MetS features, and changes in total body weight or MedDiet adherence during the follow-up (data not shown).

**Table 5:** Associations between changes in WBC counts that occurred during the follow-up and incidence of MetS.

	*OR*	*P-value*
**Leukocytes**		
Adjusted model a	1.30 (1.04–1.62)	0.021
Adjusted model 1^b^	1.32 (1.06–1.67)	0.015
Adjusted model 2^c^	1.27 (1.00–1.61)	0.050
**Neutrophils**		
Adjusted model a	1.08 (0.85–1.37)	0.532
Adjusted model 1^b^	1.06 (0.83–1.35)	0.648
Adjusted model 2^c^	1.05 (0.81–1.36)	0.702
**Lymphocytes**		
Adjusted model a	1.23 (0.99–1.52)	0.064
Adjusted model 1^b^	1.19 (0.99–1.02)	0.118
Adjusted model 2^c^	1.18 (0.94–1.49)	0.153

Odds ratios (95% confidence intervals).

Logistic regression models (outcome: MetS; independent variable: participants were dichotomized in two categories: a) those who remained in the same blood cell (leukocyte, neutrophil and lymphocyte) quartiles (2^nd^, 3^rd^, or 4^th^) or switched to a higher quartile during the follow-up; and b) those who remained in the 1^st^ quartile or switched to a lower sex-adjusted quartile during the follow-up).

a Adjusted for the intervention group, and baseline variables: age, current and former smoker, physical activity, Mediterranean diet score, alcohol BMI and recruitment node; ^b^ Model 1 plus adjusting for changes in body weight and Mediterranean diet score; ^c^ Model 2 plus adjusting for MetS components.

## Discussion

The present study is the first longitudinal assessment to have investigated the relationship between WBC counts or leukocyte subtype counts and the risk of developing MetS or some of its components in a large population sample at high cardiovascular risk. Our longitudinal assessment shows that total WBC and WBC subtype counts are directly and significantly associated with the incidence of MetS and some of its specific defining criteria. An increase in the WBC count during the follow-up was also related to a higher risk of MetS.

Our cross-sectional results are consistent with reported associations between WBC and the prevalence of MetS [18], [19]. These associations have been observed in small [14], [15], [30], [31] and large population samples [12], [18], [19], in preadolescent, adolescent and young individuals [20], [22], in healthy adults [4], [12], [19], in the elderly [30], [31], and in individuals with type 2 diabetes [5], [15], [17].

Very few of these previous cross-sectional studies have analysed the associations between WBC subtype counts and MetS. An association between total leukocyte, neutrophil and lymphocyte count and the prevalence of MetS has been observed in an elderly Chinese population [19]. This association has also been observed for total leukocyte counts and subtype counts (neutrophils, lymphocytes, monocytes, basophils and eosinophils) in a cross-sectional study of Korean individuals [18]. More recently, high circulating lymphocyte sub-population counts have been associated with an increased prevalence of MetS [21]. In our study, with the exception of basophils, all WBC subtype counts were associated with a higher prevalence of MetS.

Only two prospective studies [22], [23] have analysed the relationship between the total WBC count and the MetS (or each of its defining criteria), and both showed a direct association. However, the associations between WBC subtype counts and the incidence of MetS or its components was not specifically explored in either of these studies. The first study was conducted in a Japanese population, included 5,043 workers initially free of MetS (mean age: 42.5 years), and was followed for 7 years [23]. A total of 925 participants were newly diagnosed with MetS during follow-up. The adjusted hazard ratio for the highest versus the lowest quartile of the WBC count was 1.67 (95% CI: 1.36-2.05). The association between WBC subtypes and MetS incidence was not specifically assessed. The second study (Bogalusa cohort) was conducted in children and young adults, and it showed that there was a racial difference (the association was present in whites but not in blacks) in the correlations between WBC counts and each of the specific parameters used as defining criteria for the MetS [22]. In our study we demonstrated that the total WBC and some cell subtype counts were associated with an increased risk of MetS (and some of the defining criteria for the MetS), but we were not able to explore racial differences because the study population consisted almost exclusively of Europeans. We found that WBC counts were also positively associated with three of the parameters used as defining criteria for the MetS: hyperglycemia, low HDL-cholesterol, and hypertriglyceridemia.

The positive association observed in our study between the total leukocyte, neutrophil and lymphocyte counts and the hyperglycemia component was supported by a meta-analysis of 20 cross-sectional and prospective cohort studies exploring the association between WBC counts and type 2 diabetes [5]. The biological mechanisms explaining these associations remain to be elucidated. It seems very likely that a low-grade systemic inflammation associated with obesity and insulin resistance precedes and predicts the development of diabetes. Adipocytes, especially in obese subjects, secrete a number of proinflammatory cytokines, [32] some of which have been shown to directly inhibit insulin signalling [33]. Adipocytokines probably act through master proinflammatory regulators such as those of the nuclear factor-κB and the c-Jun NH_2_-terminal kinase (JNK)/AP-1 signalling pathways [34] to modulate the expression of gene coding for many inflammatory proteins and to alter insulin signalling.

The prospective association observed in our study between the total and subtype WBC counts and hypertriglyceridemia or low-HDL cholesterol components of MetS is in agreement with previous cross-sectional studies and the prospective assessment in the Bogalusa cohort. This can also be explained through inflammation. Early cross-sectional studies conducted in hyperlipidemic patients revealed that an increase in the total peripheral leukocyte count and subtype counts (except for eosinophils) was mainly associated with higher serum triglyceride concentrations and hypertriglyceridemia [35]. Other epidemiological studies have also reported a positive association between WBC count (or some subtype counts) and hypertriglyceridemia or low-HDL cholesterol concentrations [35], [36]. An association between total lymphocyte counts and hypertriglyceridemia or low-HDL cholesterol has also recently been reported only in men [36].

Lipid metabolism disorders found during inflammation are the result of increased triglyceride concentrations and decreased HDL-cholesterol levels (two components of the so-called atherogenic dyslipidemia). Inflammation and atherogenic dyslipidemia may be linked by the fact that TNF alpha and IL-6 stimulate lipolysis and increase the flow of free fatty acids to the liver. This increase in free fatty acids induces hepatic triglyceride synthesis and increases very low density lipoprotein secretion from the liver, both of which increase hepatic triglyceride production and secretion, thus causing hypertriglyceridemia [37]. TNF alpha and IL-6 also suppress lipoprotein lipase synthesis in adipose tissue, which may contribute to hypertriglyceridemia and the low HDL-cholesterol concentrations observed in individuals with intra-abdominal obesity [38].

Some authors have hypothesized that a high WBC count causes a low-grade chronic inflammation that impairs endothelial function. This affects the production of nitric oxide and prostacyclin and results in hypertension and the loss of vasodilator capacity [39]. In our study, the lack of association between the leukocyte count and high blood pressure may be explained by the fact that more than 90% of our study participants were hypertensive, thus making it difficult to explore these associations.

The current study has some limitations. First, the high prevalence of cardiovascular risk factors among our participants may limit the generalizability of our findings to general populations or younger adults. However, this high prevalence may even increase the interest of the association reported. It is well known that the wider the between-subject variability in a biomarker, the easier it is to find associations between that biomarker and the outcome. The homogeneity of the high cardiovascular risk of the PREDIMED participants probably decreases the likelihood of finding strong associations with factors potentially associated with the characteristics of the MetS, because the between-subject variability is lower than in a representative sample of the general population. Therefore, the fact that we have found an association between leukocyte counts and MetS even in our suboptimal scenario may be seen as support for that association. Second, we did not exclude from the analysis those participants with a recognized chronic inflammatory disease, which may influence the WBC count. However, we did explore all these associations before or after excluding participants with a WBC count ≥11.0×10^9^ cells/L, and the results remained essentially the same (Appendix S1). Third, we admit that the true statistical model relating the measured variables cannot really be known and standard logistic regression and other similar models have been defined only as “simplified pictures of reality” [40]. However, we believe that the use of this simplified approach has become familiar to the scientific community because it is the standard approach in epidemiology. Fourth, the inclusion of WBC values increased the area under the ROC curve only slightly and non-significantly. Finally, the prospective component was only a modest proportion of the total trial, which may lead to selection bias.

## Conclusions

Total and subtype WBC counts were directly and significantly associated with the incidence of MetS and also with the hypertriglyceridemia, low HDL-cholesterol and high fasting plasma glucose components of the MetS. WBC counts can be regarded as markers of an increased production of cytokines and acute-phase reactants, and the activation of the inflammatory signalling network involved in the pathogenesis of insulin resistance and atherogenic dyslipidemia, the main metabolic disorders underlying MetS [41].

## Supporting Information

Appendix S1 **Odds Ratios for the MetS according to the leukocyte count measured 2 years previously.**
(DOC)
