# Supplementary material for: White Blood Cell Counts as Risk Markers of Developing Metabolic Syndrome and Its Components in the Predimed Study
Source: PLoS One. 2013 Mar 19;8(3):e58354. doi: 10.1371/journal.pone.0058354 (PMC3602299; doi:10.1371/journal.pone.0058354)
Supplement: Appendix S1 — Odds Ratios for the MetS according to the leukocyte count measured 2 years previously. (DOC) [file pone.0058354.s001.doc]

**SUPORTING INFORMATION LEGENDS**

**Appendix S1.**

Odds Ratios for the MetS according to the leukocyte count measured 2 years previously

| Odds ratios for an increment of +2*10^9^ cells/L | All participants | Excluding those with leukocyte>11*10^9^ cells/L |
| --- | --- | --- |
| Sex-, age-adjusted | 1.22 (1.10-1.36) | 1.25 (1.10-1.42) |
| Multivariable-adjusted | 1.20 (1.08-1.34) | 1.24 (1.09-1.41) |
| Fully-adjusted model^b^ | 1.17 (1.05-1.31) | 1.19 (1.05-1.36) |

^a^Generalized estimating equations adjusted for intervention group, and baseline age, tobacco use, physical activity, Mediterranean diet score and alcohol consumption, BMI and recruitment centre.

^b^Generalized estimating equations adjusted for variables used in ^a^ plus baseline features of the MetS.
